# The role of DNA mismatch repair status in oesophagogastric cancer treated with perioperative chemotherapy: evidence from the MRC ST03 and OE05 trials

**DOI:** 10.1038/s41416-026-03594-9

**Published:** 2026-08-25

**Authors:** Panagiotis Ntellas, Tom Lund, Georgina A. Keogh, Manuel Salto-Tellez, Katharina von Loga, Tatiany Silveira, Susanna Riisnaes, Jon Laye, Ruth E. Langley, Matthew G. Nankivell, William H. Allum, Avani Athauda, David Cunningham, Heike I. Grabsch

**Affiliations:** 1 Department of Gastrointestinal Medical Oncology, Royal Marsden Hospital, London and Surrey, UK; 2 The Integrated Pathology Unit, The Institute of Cancer Research & The Royal Marsden Hospital, Sutton, UK; 3 The Precision Medicine Centre, Queen’s University Belfast, Belfast, UK; 4 Waiv, Paris, France; 5 Pathology and Data Analytics, Leeds Institute of Medical Research at St. James’s, University of Leeds, Leeds, UK; 6 UCL Innovative Clinical Trials Unit, University College London, London, UK; 7 Department of Oncology and Department of Surgery, Royal Marsden NHS Foundation Trust, London, UK; 8 Department of Pathology, GROW - Research Institute for Oncology and Reproduction, Maastricht University Medical Center +, Maastricht, Netherlands

**Keywords:** Gastric cancer, Oesophageal cancer, Tumour biomarkers

## Abstract

**Background:**

Mismatch repair deficiency (MMRd) is a key determinant of tumour biology, however, its prognostic significance in patients with resectable oesophagogastric adenocarcinoma treated with perioperative cytotoxic chemotherapy remains uncertain.

**Methods:**

MMR status was assessed in 1424 patients with oesophagogastric adenocarcinoma enroled in the MRC OE05 and ST03 trials, which investigated perioperative chemotherapy regimens. Associations between MMR status, clinicopathological variables, overall survival (OS), progression-free survival (PFS) and post-progression survival (PPS) were investigated.

**Results:**

In total, 78 (5.5%) tumours were MMRd, 80.8% due to MLH1 loss. Patients with MMRd tumours had improved OS compared with patients with MMR proficient (MMRp) tumours (median unreached vs 28.0 months; *p* = 0.0009; 5-year rates 50.1% vs. 34.3%), and PFS (median 56.7 vs 21.4 months; *p* = 0.0032). On multivariable analysis, MMRd remained associated with improved OS (HR 0.60, 95% CI 0.42–0.87; *p* = 0.007) and PFS (HR 0.64, 95% CI 0.45–0.90; *p* = 0.011). PPS did not differ by MMR status.

**Discussion:**

This is the first study to suggest that perioperative cytotoxic chemotherapy does not abrogate the favourable prognostic impact of MMRd prior to disease progression. However, MMR status did not influence survival after progression. Our results challenge clinical concerns that cytotoxic chemotherapy may be detrimental in patients with resectable MMRd oesophagogastric adenocarcinoma.

## Introduction

The standard management of patients with resectable oesophagogastric adenocarcinoma is multimodal as landmark trials have shown that perioperative or neoadjuvant cytotoxic chemotherapy improves survival compared to surgery alone [1–5]. However, treatment selection remains guided by clinical and pathological factors, with limited integration of molecular biomarkers such as the DNA mismatch repair (MMR) status [6, 7].

The MMR system preserves genomic integrity by correcting DNA replication errors [8]. Loss of function of one or more MMR proteins (MLH1, MSH2, MSH6, PMS2) results in a hypermutated tumour phenotype characterized by microsatellite alterations and increased mutational burden driving a distinct pattern of tumour evolution compared with MMR proficient (MMRp) cancers [9, 10]. MMR deficiency (MMRd) leads to microsatellite instability (MSI) and defines a biologically and clinically distinct tumour subtype across multiple tumour types, including endometrial, colorectal and other gastrointestinal (GI) malignancies [10–12]. In several tumour types, MSI/MMRd is associated with improved prognosis; notably, in patients with early-stage colorectal cancer, MSI/MMRd predicts lack of benefit from adjuvant fluoropyrimidine monotherapy [13–17].

In the upper GI tract, MSI/MMRd tumours are most frequent in the stomach, less common at the oesophagogastric junction and rare in the oesophagus [13–16]. However, reported MSI/MMRd frequencies in oesophagogastric cancer vary widely from 4% to 24% reflecting heterogeneity in primary tumour location, disease stage, patient demographics and assay methodology [14, 15, 17, 18]. In the perioperative setting including major trials such as MAGIC [2], CLASSIC [19], ARTIST [20], and ITACA-S [21], MSI/MMRd occurred in 6% to 10% of tumours [13]. MMRd tumours are more often associated with older age, female sex, intestinal-type histology, fewer lymph node metastases and earlier disease stage at diagnosis, however, the strength and consistency of these associations differ across studies [6, 13–15, 22].

Available data suggest that patients with resectable MMRd oesophagogastric adenocarcinoma may have a better prognosis than those with MMRp oesophagogastric cancers [13, 23]. However, the prognostic evidence is inconsistent across studies [24], and the relationship between MMR status and benefit from cytotoxic chemotherapy is much less clear in patients with MMRd oesophagogastric cancer [15, 25]. These uncertainties reflect limitations of the existing literature, which is based on retrospective observational studies and meta-analyses, often constrained by small numbers of MMRd tumours, heterogeneous patient populations and variability in MMR/MSI testing methodology [13, 15, 25].

Despite a strong biological rationale and emerging clinical data, high-quality prospective evidence evaluating the prognostic relevance and possible differential sensitivity to standard perioperative cytotoxic chemotherapy regimens of MMRd in patients with resectable oesophagogastric adenocarcinomas treated with cytotoxic chemotherapy remains scarce.

To address this important clinical knowledge gap, we performed a retrospective analysis of the MMR status in tumour specimens from patients enroled in the UK MRC ST03 and OE05 trials—two large, multicentre, randomized studies evaluating different neoadjuvant or perioperative cytotoxic chemotherapy regimens in patients with resectable oesophagogastric adenocarcinomas [26, 27]. By leveraging these well-characterised trial cohorts with long-term follow-up and centrally reviewed pathology, we aimed to provide robust, prospectively collected evidence on the prognostic significance of MMR status in patients with oesophagogastric cancers treated with perioperative cytotoxic chemotherapy.

## Materials & methods

### Clinical trial cohorts

The UK MRC OE05 trial was a phase III study randomising patients with resectable oesophageal or junctional adenocarcinoma to either four cycles of epirubicin, cisplatin, and capecitabine (ECX) followed by surgery or two cycles of cisplatin and fluorouracil (CF) followed by surgery. The trial was designed to evaluate whether intensifying pre-operative cytotoxic chemotherapy improves outcomes. There was no significant overall survival advantage for the more intensive treatment approach [27].

The UK MRC ST03 was a phase II/III trial randomising patients with resectable gastric or junctional adenocarcinoma to perioperative ECX chemotherapy with or without bevacizumab followed by surgery. There was no significant overall survival advantage from adding bevacizumab [26].

### Study population and data collection

Between January 2005 and October 2011, 897 patients were randomised in the OE05 trial, and 1063 patients were randomised in the ST03 trial between October 2007 and March 2015. Tissue suitable for mismatch repair (MMR) testing was available from 1424 patients. Individual patient-level data were retrieved from databases held by the MRC Clinical Trials Unit at University College London, UK. Pathology was centrally reviewed as part of the clinical trial. Part of the results from this study were presented as a poster at the GI ESMO Meeting in Barcelona in 2025, the abstract is published in the July 2025 supplement in Annals of Oncology. The translational analysis of the MRC OE05 and ST03 trials was approved by South West—Cornwall & Plymouth Research Ethics Committee (REC reference: 18/SW/0279, IRAS Reference 257378) and the South East Research Ethics Committee (REC reference: 07/H1102/111, IRAS Reference 144220). The South East Research Ethics Committee waived the need for informed consent. The study was performed in accordance with the Declaration of Helsinki.

### Assessment of DNA mismatch repair status

MMR status was determined by assessing protein expression of MLH1, MSH2, MSH6 and PMS2 in formalin-fixed, paraffin-embedded tumour tissue samples using brightfield single protein immunohistochemistry (IHC) of consecutive tissue sections in 497 patients (35%) and multiplex immunofluorescence (mIF) on a single tissue section in 927 patients (65%).

For IHC, tissue sections were stained using an automated staining platform (BOND 3.0). All primary antibodies were used at a dilution of 1:50: MLH1 (mouse monoclonal, clone ES05, Dako), MSH2 (mouse monoclonal, clone FE11, Dako), MSH6 (rabbit monoclonal, clone EP49) and PMS2 (rabbit monoclonal, clone EP51) followed by polymer reagent, 3,3′-Diaminobenzidine (DAB) chromogen and Haematoxylin counterstain. Stained slides were scanned at ×40 magnification with an Aperio or Hamamatsu scanner for digital MMR scoring by two experienced gastrointestinal pathologists (HIG and MT).

For mIF, a panel of five biomarkers [4 MMR and pan-cytokeratin (panCK)] plus 4′,6-diamidino-2-phenylindole (DAPI) was designed and subsequently optimised. The Opal-TSA system was adopted using the Opal Polaris 7-Colour Automation IHC Kit (Quaternix, formerly Akoya Biosciences). After Opal fluorophore deposition, the slides underwent subsequent rounds of antigen retrieval to remove the primary/secondary antibody complexes, preparing the tissue section for the next antibody. This process was repeated until all markers were labelled as per manufacturer’s instructions. Finally, spectral DAPI (Quaternix, formerly Akoya Bioscience) was added, and the slides were mounted in X6 Immu-Mount (Fisher Scientific). Slides were scanned by the PhenoImager HT 2.0 multispectral imaging platform (Quaternix—formerly Akoya Biosciences) using PhenoImager HT 2.0 software. For details on primary antibodies and mIF staining conditions, see Supplementary Table S1. The mIF slides were analysed using the HALO Image Analysis Platform version 3.6.4134 (Indica Labs) to segment cells and identify staining in the nucleus. Subsequently, an expert gastrointestinal pathologist reviewed all mIF stained tissue sections manually to determine whether nuclei are positive for the respective marker or not. AI-based image analysis was not used to determine the final expression status.

DNA mismatch repair (MMR) status evaluation was done following the Royal College of Pathology guidelines, and the same criteria were applied for both brightfield IHC and mIF: Nuclear staining of MLH1/MSH2/MSH6/PMS2 in any number of tumour cells was classified as positive expression, while loss of nuclear staining in all tumour cells in the presence of positive staining in stromal cells or lymphocytes (internal controls), was classified as loss of expression. A tumour was classified as DNA mismatch repair proficient (MMRp) if all four proteins were expressed. Loss of expression of a single MMR protein was sufficient to classify a tumour as DNA mismatch repair deficient (MMRd). As this is the first published instance that mIF technology was deployed to determine MMR protein expression, all cases classified as MMRd based on mIF were subsequently subjected to single protein brightfield IHC for confirmation. IHC and mIF data showed 100% concordance in determining the MMR status. Owing to resource constraints, we were not able to perform conventional IHC on all tumours classified as MMRp by mIF.

### Statistical analysis

This research study was designed and reported in accordance with the REMARK (Reporting Recommendations for Tumour Marker Prognostic Studies) guidelines [13], see details in Supplementary Tables S2 and S3. The primary objective of the study was to evaluate the association between MMR status and overall survival (OS); secondary objectives included progression-free survival (PFS), post-progression survival (PPS), and assessment of survival stratifying patients by MMR protein loss phenotype (patients were stratified into four distinct subgroups based on protein loss: any MLH1 loss, isolated PMS2 loss, isolated MSH2 and/or MSH6 loss and MMRp). PFS was defined as time from randomisation to first progression or death from any cause, with patients alive and progression-free censored at last follow-up. PPS was defined as the time from first progression to death.

Variables considered in the analyses included: type of progression (local only vs distant); surgery status (resection performed vs no resection performed); trial cohort (ST03 vs OE05); clinical characteristics at time of diagnosis: depth of invasion (cT1-2 vs cT3-4), lymph node status (cN0 vs cN + ), endoscopic tumour location at time of diagnosis (oesophagus vs gastro-oesophageal junction [GOJ] vs stomach), ECOG performance status (PS, 0 vs 1), treatment arm (neoadjuvant CF vs neoadjuvant ECX vs perioperative ECX vs perioperative ECX + bevacizumab); characteristics derived from the pathological evaluation of the resection specimen: ypT0-2 vs ypT3-4, ypN0 vs ypN1 + , resection margin status (R0 vs R1), presence of lymphovascular invasion (LVI No vs Yes), Mandard primary tumour regression grade (TRG, 1 and 2 vs 3, 4 and 5) [28]. Descriptive statistics were presented as counts and percentages for categorical variables and as medians with interquartile ranges for continuous variables.

The relationship between MMR status, clinicopathological variables including patterns of progression was analysed using binary logistic regression. Odds ratios (ORs) were calculated by exponentiating the regression coefficients, and *p* values were derived from the Wald test. For continuous variables, non-parametric methods were used as appropriate.

Time-to-event outcomes were analysed using Kaplan-Meier curves, median survival time, 5-year survival and 95% confidence intervals (CIs) were reported; group comparisons were assessed by log-rank statistics. Univariable Cox proportional hazards model was used to assess the relationship between MMR status and OS, PFS or PPS. A multivariable Cox proportional hazards model was used to assess whether MMR status provides prognostic information after adjustment for clinically relevant covariates. Pre-operative and post-operative clinicopathological factors were evaluated in separate models to reduce overfitting and to account for potential interdependence between clinical characteristics at the time of diagnosis and pathological factors at the time of resection. This approach allowed us to explore whether the prognostic association of MMR status persists when accounting for baseline clinical factors and whether it remains robust after adjustment for pathological features known only post-surgery. All covariates were prespecified based on clinical relevance. Subgroup analyses examined the effect of the MMR status on survival outcomes across key clinical and pathological strata. Two-sided p-values < 0.05 were considered statistically significant. All analyses were performed using the R- project statistical software (version 4.5.1).

## Results

**Fig. 1:**
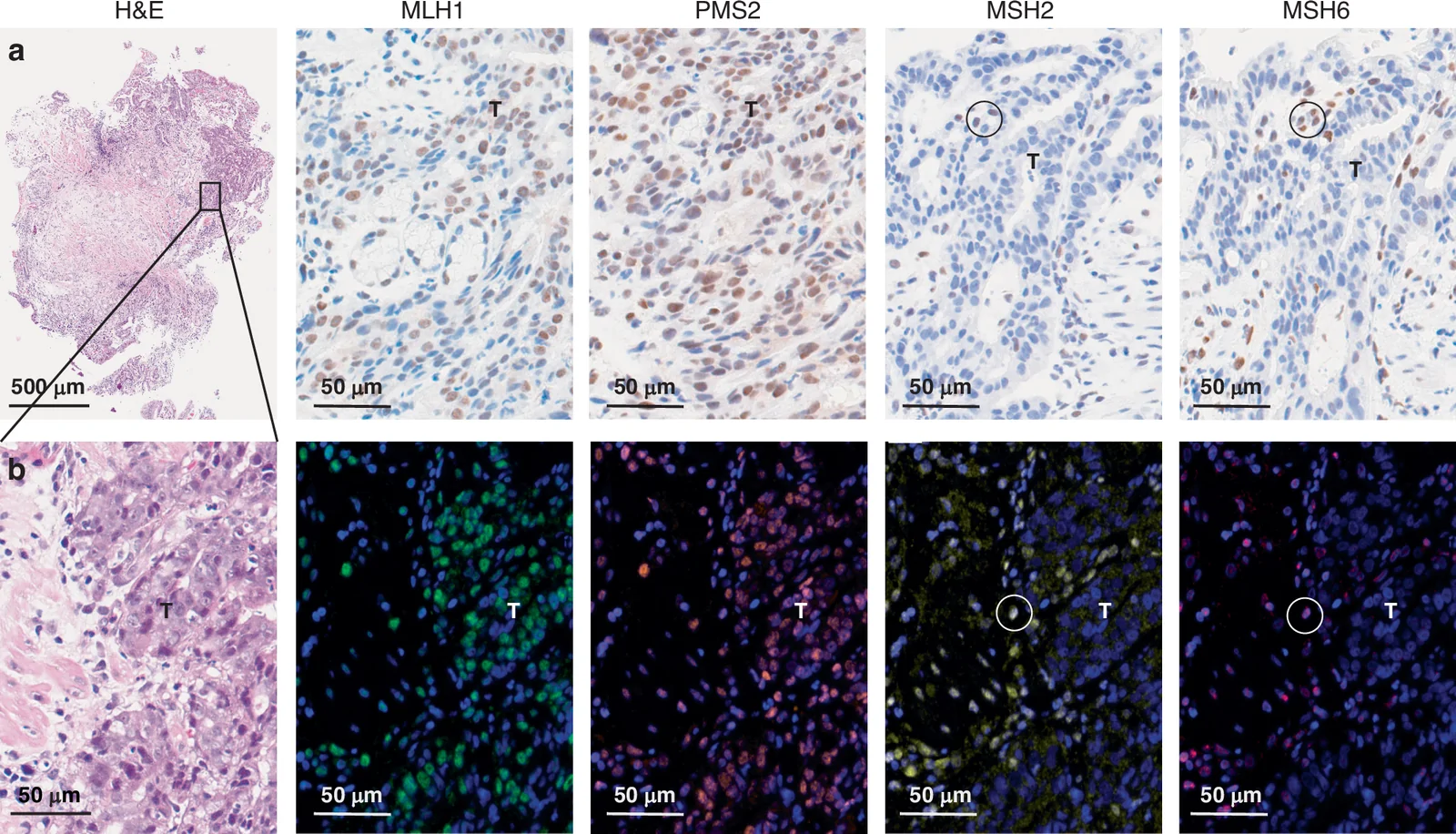
Immunohistochemistry and multiplex immunofluorescence staining of DNA mismatch repair proteins. **a** Pre-treatment biopsy, Haematoxylin and Eosin (H&E) stained, low magnification overview. **b** Higher magnification of (**a**) with adenocarcinoma, intestinal-type, H&E stained, T = adenocarcinoma. Top row: Consecutive tissue sections stained with immunohistochemistry for MMR proteins (MLH1, PMS2, MSH2, MSH6) using DAB as chromogen (brown), Haematoxylin counterstain (blue). The adenocarcinoma is positive for MLH1 and PMS2; negative for MSH2 and MSH6 (circle highlights positive internal controls). T = adenocarcinoma. Bottom row: Single tissue section from the same tissue block stained for MLH1, PMS2, MSH2 and MSH6 with multiplex immunofluorescence using different fluorophores and DAPI counterstain of nuclei in blue. The adenocarcinoma is positive for MLH1 and PMS2; negative for MSH2 and MSH6 (circle highlights positive internal controls). T = adenocarcinoma.

DNA mismatch repair (MMR) status was determined in 1424 tumours using brightfield single protein immunohistochemistry in 497 tumours (35%) and using multiplex immunofluorescence (mIF) in 927 (65%) (Fig. 1). Overall, 78 (5.5%) tumours were MMR deficient (MMRd) and 1346 (94.5%) were MMR proficient (MMRp). Among MMRd tumours, 63 (80.8%) showed loss of MLH1 expression alone or in combination with PMS2 loss, 3 (3.8%) showed isolated loss of PMS2 expression and 12 (15.4%) loss of MSH2 and/or MSH6 expression.

### Relationship between MMR status and clinicopathological variables

**Fig. 2:**
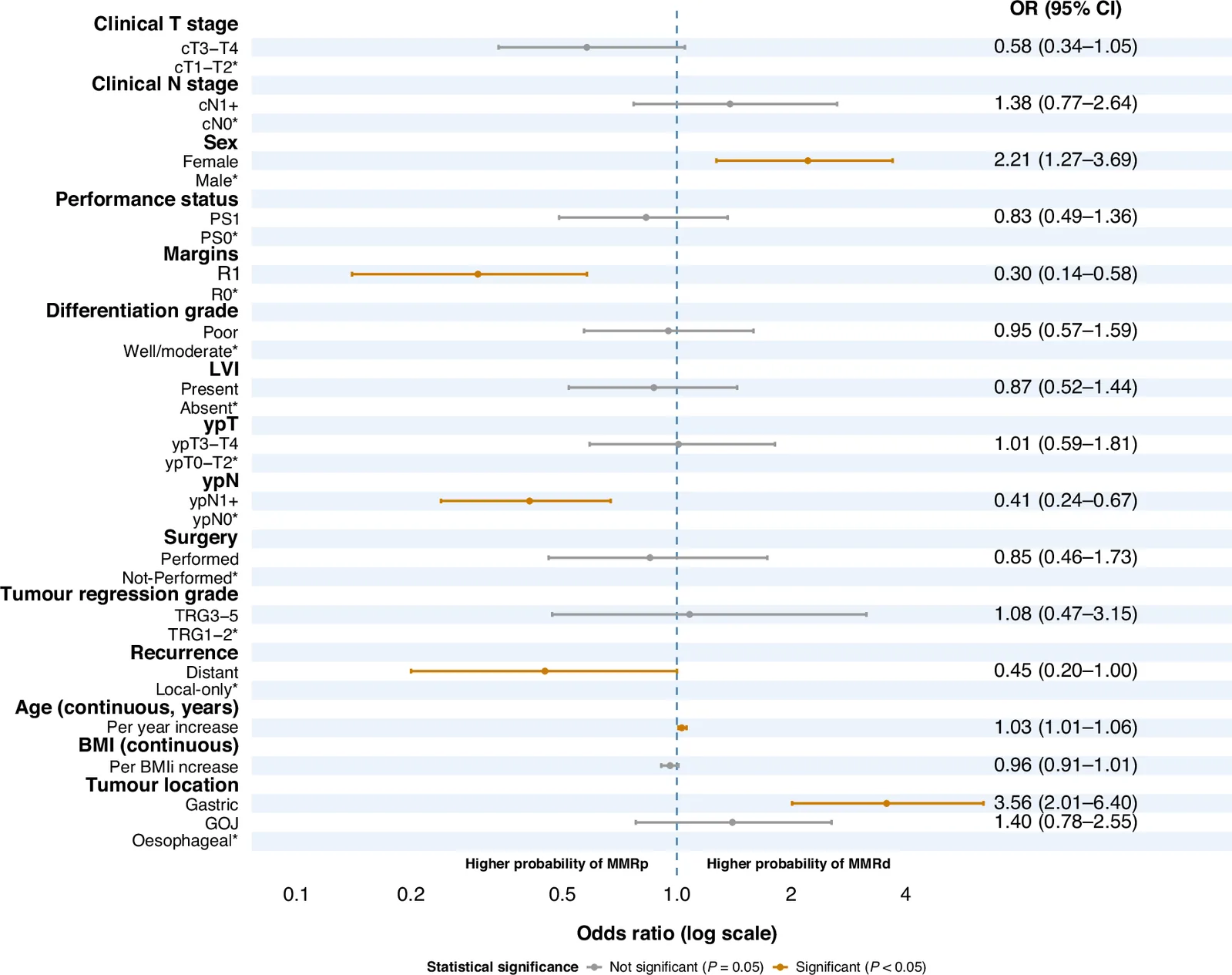
Univariable analysis of clinicopathological factors and MMR status. The Forest plot illustrates odds ratios (OR) with 95% confidence intervals for clinicopathological variables assessed using univariable binary logistic regression. OR > 1 indicates higher probability of MMR deficiency (MMRd); OR < 1 indicates higher probability of MMR proficiency (MMRp). Red bars represent statistically significant associations (*p* < 0.05); grey bars indicate non-significant results (*p* ≥ 0.05). *Reference category. T depth of tumour invasion, N lymph node status, LVI lymphovascular invasion, R1 resection margin positive, R0 resection margin negative, BMI body mass index, GOJ gastroesophageal junction.

Clinicopathological characteristics stratified by MMR status are summarised in Table 1. Patients with MMRd tumours were older, more frequently female (OR 2.21, 95% CI 1.27–3.69; *p* = 0.004) and more likely to have tumours located in the stomach rather than the oesophagus (OR 3.56, 95% CI 2.01–6.40; *p* < 0.001). In resection specimens, patients with MMRd tumours had 59% lower odds of lymph node metastasis (ypN1 + : OR 0.41, 95% CI 0.24–0.67; *p* = 0.001), and 70% lower odds of R1 resection (OR 0.30, 95% CI 0.14–0.58; *p* = 0.001). Patients with MMRd tumours showed a relative predominance of local rather than distant disease progression, with a trend towards 55% lower odds of distant tumour progression compared to patients with MMRp tumours (OR 0.45, 95% CI 0.2–1.00; p = 0.049). No significant associations were observed between MMR status and clinical T or N stage at diagnosis, post-surgery T stage (ypT), Mandard primary tumour regression grade (TRG), histological grade of differentiation, presence of lymphovascular invasion (LVI), or Eastern Cooperative Oncology Group (ECOG) performance status. The effect sizes and associations between MMR status and clinicopathological variables are illustrated in Fig. 2.

**Table 1:** Patient characteristics.

	Total^#^ (*n* = 1424)		MMR proficient (*n* = 1346, 94.5%)		MMR deficient (*n* = 78, 5.5%)	
	*n*	%	*n*	%	*n*	%
**Trial**						
OE05	736	51.7	710	96.5	26	3.5
ST03	688	48.3	636	92.4	52	7.6
Treatment arm*						
Pre-op CF	381	26.8	367	96.3	14	3.7
Pre-op ECX	355	24.9	343	96.6	12	3.4
Peri-op ECX	352	24.7	328	93.2	24	6.8
Peri-op ECX + Bevavicumab	336	23.6	308	91.7	28	8.3
Performance status*						
PS 0	970	68.1	914	94.1	56	5.8
PS 1	454	31.9	432	95.2	22	4.8
**Sex**						
Male	1222	85.8	1164	95.3	58	4.7
Female	202	14.2	182	90.1	20	9.9
Age*						
Median [IQR] years	63 [56, 68]		62 [56, 67]		65 [58, 70]	
Body Mass Index*						
Median [IQR]	26 [24, 29]		27 [24, 30]		26 [24, 29]	
Tumour location*						
Oesophagus	622	45.6	600	96.5	22	3.5
Gastroesophageal junction	356	26.1	336	94.4	20	5.6
Stomach	385	28.2	352	91.4	33	8.6
Depth of invasion (cT)*						
1	52	3.7	44	84.6	8	15.4
2	152	10.8	143	94.1	9	5.9
3	1154	81.7	1097	95.1	57	4.9
4	55	30.5	51	92.7	4	7.3
Lymph node status (cN)*						
0	301	21.3	288	95.7	13	4.3
1+	1112	78.7	1047	94.2	65	5.8
**Surgery performed**						
No	176	12.4	165	93.8	11	6.3
Yes	1246	87.6	1179	94.6	67	5.4
**Depth of invasion (ypT)**						
0	41	3.3	38	92.7	3	7.3
1	128	10.4	123	96.1	5	3.9
2	175	14.2	165	94.3	10	5.7
3	721	58.6	680	94.3	41	5.7
4	166	13.5	160	96.4	6	3.6
**Lymph node status (ypN)**						
0	444	36.0	407	91.7	37	8.3
1+	788	64.0	760	96.4	28	3.6
**Primary tumour regression grade**^**+**^						
1	42	3.2	39	92.9	3	7.1
2	61	4.6	59	96.7	2	3.3
3	214	16.2	205	95.8	9	4.2
4	563	42.5	533	94.7	30	5.3
5	444	33.5	419	94.4	25	5.6
**Resection margin status**						
Negative (R0)	773	63.4	719	93.0	54	7.0
Positive (R1)	447	36.6	437	97.8	10	2.2
**Grade of differentiation**						
Well/moderate	534	45.5	505	94.6	29	5.4
Poor	639	54.5	606	94.8	33	5.2
**Lymphovascular invasion**						
No	633	52.2	598	94.5	35	5.5
Yes	580	47.8	552	95.2	28	4.8
**Type of Progression**						
Local progression only	193	17.2	181	93.8	12	6.22
Distant metastasis only	281	25.2	271	96.4	10	3.6
Distant met. + local progression	171	15.4	168	98.2	3	1.8
No progression	469	42.2	430	91.7	39	8.3

# numbers for individual variables do not always add up to the total number due to missing values, *at time of randomisation, + according to Mandard, *CF* cisplatin + 5-fluorouracil, *ECX* epirubicin + cisplatin + capecitabine, *IQR* interquartile range.

### Relationship between MMR status and survival

**Fig. 3:**
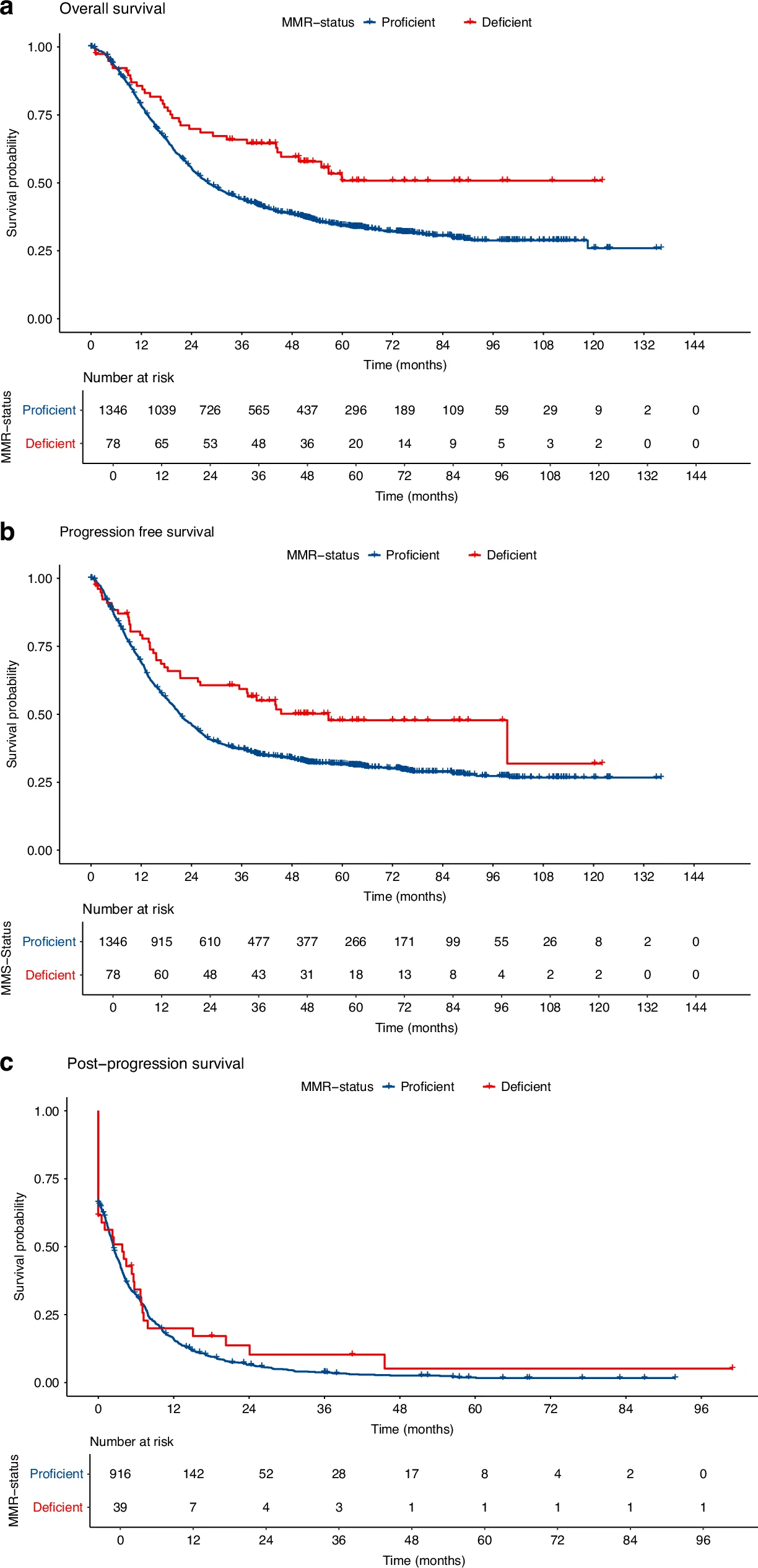
Kaplan-Meier curves for overall survival, progression free survival and time from first progression to death stratified by MMR status. **a** Patients with MMR deficient tumours have significantly improved overall survival compared to those with MMR proficient tumours (log-rank *p* = 0.00094). **b** Patients with MMR deficient tumours have significantly improved progression free survival compared to those with MMR proficient tumours (log-rank *p* = 0.0032). **c** The Kaplan-Meier plot shows that mortality is high after first progression. Patients with MMR deficient tumours have similar post-progression survival compared to those with MMR proficient tumours (log-rank *p* = 0.31).

Median follow-up time from randomisation was 64 months. Kaplan-Meier curves for OS, PFS and PPS by MMR status are shown in Fig. 3. MMR status was significantly associated with both OS and PFS (*p* = 0.0009 and *p* = 0.0032, respectively). Median OS was 28.0 months (95% CI 25.8–31.3 months) for patients with MMRp tumours, whereas it was not reached for those with MMRd tumours; 5-year OS rates were 34.3% (95% CI 31.8–37.1%) and 50.8% (95% CI 39.7–65.0%), respectively. Median PFS was 21.4 months (95% CI 19.96–23.14) in patients with MMRp tumours and 56.7 months (95% CI, 35.40%—not reached) in patients with MMRd tumours; 5-year PFS rates were 31.6% (95% CI 29.2–34.3%) and 47.8% (95% CI 37.2–61.3%), respectively. On univariable analysis, MMR deficiency was associated with a 42% lower risk of death (HR 0.56; 95% CI 0.40–0.79; *p* = 0.001) and improved PFS (HR 0.62; 95% CI 0.45–0.85; *p* = 0.004).

We explored post-progression survival (PPS) according to MMR status, see Fig. 3. The Kaplan-Meier plot showed a rapid decline in survival immediately following disease progression for both groups, reflecting high early mortality. The survival curves remained overlapping, with no significant difference in survival by MMR status (log-rank *p* = 0.31).

To determine whether the favourable prognosis of patients with MMRd tumours was driven by the loss of a specific MMR protein, patients were stratified by protein loss pattern in the primary tumour (MLH1 ± PMS2, PMS2 alone, MSH2 and/or MSH6, Supplementary Fig. 1). Patients with tumours demonstrating MLH1 loss had superior outcomes, with median OS not reached (5-year OS rate: 55.9%, 95% CI 43.3–72.0) and a median PFS of 99 months (5-year PFS rate: 51.3%, 95% CI 39.3–66.8). In contrast, patients with tumours demonstrating MSH2/MSH6 loss had poorer outcomes (median OS 29 months, 5-year OS rate: 33.3%, 95% CI 15.0–74.2; median PFS 26 months, 5-year PFS rate: 33.3%, 95% CI 15.0–74.2). OS and PFS curves from patients with tumours demonstrating MSH2 and/or MSH6 loss broadly overlap with those from patients with MMRp tumours. Patients with tumours demonstrating isolated PMS2 loss also appeared to have poorer outcomes, however, this finding should be interpreted with caution given the very small number of patients in this subgroup.

### Survival subgroup analyses

**Fig. 4:**
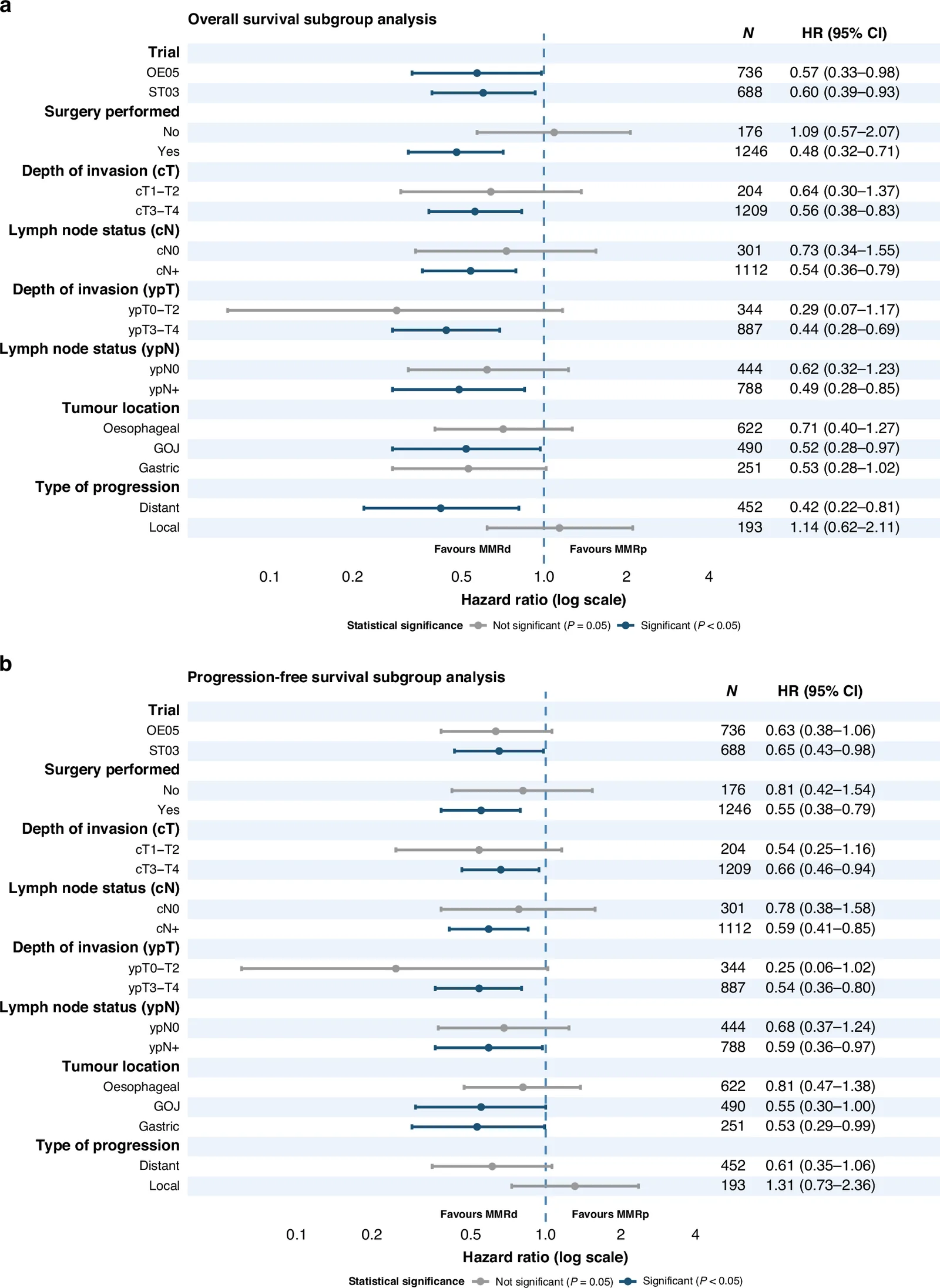
Relationship between MMR status and survival within distinct clinicopathological subgroups. **a** Univariable overall survival analysis. **b** Univariable progression-free survival analysis. Blue bars represent statistically significant associations (*p* < 0.05); grey bars indicate non-significant results (*p* ≥ 0.05). HR hazard ratio, CI confidence interval, GOJ gastroesophageal junction, MMRd mismatch repair deficient, MMRp mismatch repair proficient.

Overall, patients with MMRd tumours showed improved OS and PFS across most analysed subgroups (Fig. 4). The survival advantage of patients with MMRd tumours was consistent in both the OE05 and ST03 trials. Among patients who underwent surgery, tumour MMR deficiency was associated with significantly longer OS (HR 0.48, 95% CI 0.32–0.71) and PFS (HR 0.55, 95% CI 0.38-0.79), whereas no significant difference by MMR status was seen in OS or PFS in patients who did not undergo surgery (OS p = 0.798, PFS *p* = 0.519). Improved OS and PFS were also observed in patients with MMRd tumours and more advanced T or N stage, whether assessed clinically at diagnosis or pathologically in the resection specimen. Patients with MMRd tumours and distant progression had a significantly better OS compared to those with MMRp tumours (OS HR 0.42, 95% CI 0.22–0.81, *p* = 0.01). OS did not differ by MMR status in patients with only local progression (*p* = 0.665).

### Multivariable survival analysis

#### Pre-operative model

**Fig. 5:**
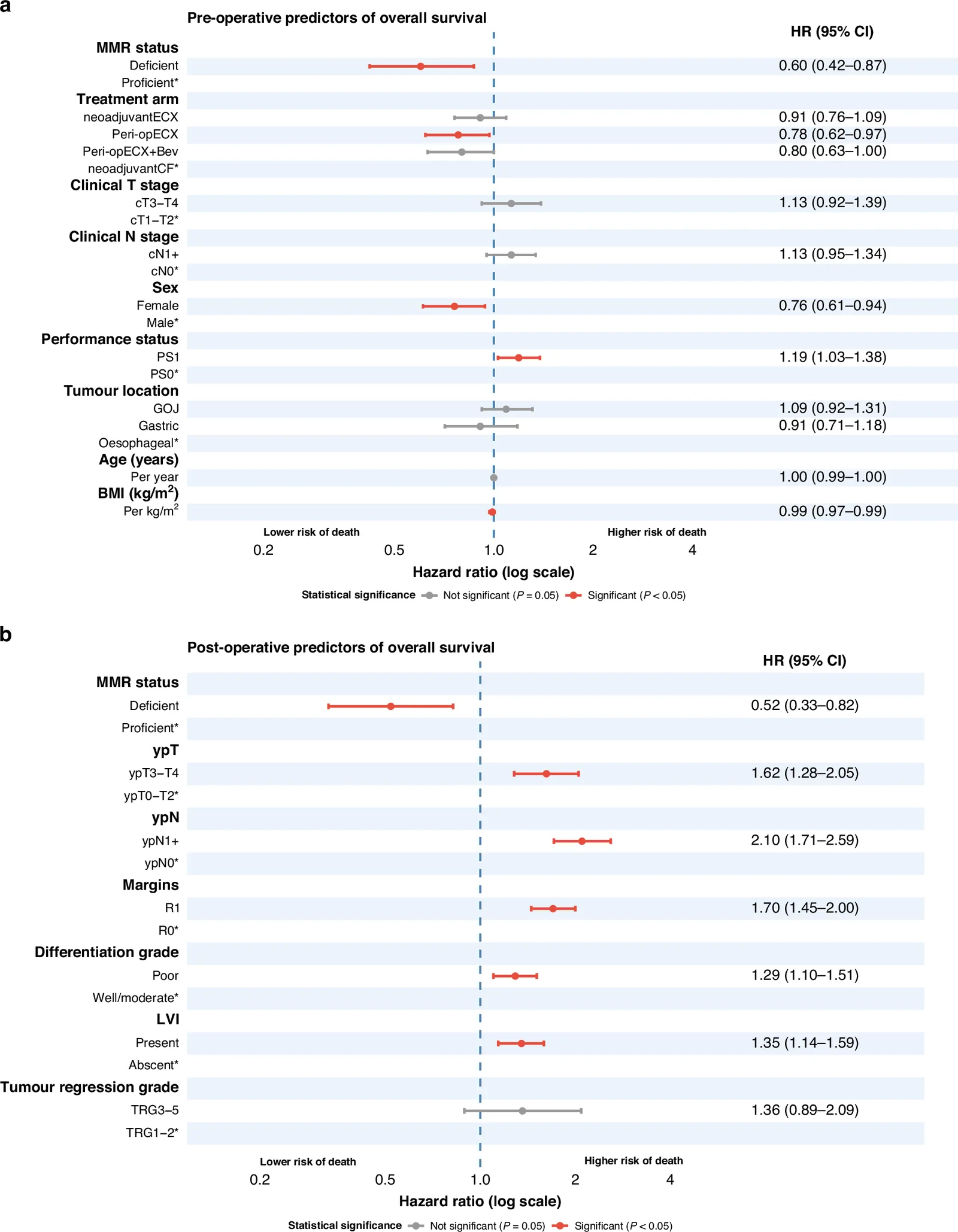
Multivariable analyses of pre- and post-operative predictors of overall survival. **a** Pre-operative predictors of overall survival. **b** Post-operative predictors of overall survival. Hazard ratio (HR) < 1 indicates lower risk of death or progression, HR > 1 indicates higher risk. Red bars represent statistically significant associations (*p* < 0.05); grey bars indicate non-significant results (*p* ≥ 0.05). *Reference category. ECX epirubicin, cisplatin and capecitabine, Bev bevacizumab, CF cisplatin and 5-fluorouracil, T depth of invasion, N lymph node status, GOJ gastroesophageal junction, BMI body mass index, R1 resection margin positive, R0 resection margin negative, LVI lymphovascular invasion, TRG tumour regression grade of the primary tumour according to Mandard.

In the multivariable model incorporating pre-operative factors (sex, BMI, age, primary tumour location, cT, cN, performance status, treatment arm, and MMR status), MMR deficiency remained independently associated with improved outcomes, conferring a 40% lower risk of death (OS HR 0.60; 95% CI 0.42–0.87; *p* = 0.007) and a 36% lower risk of progression (PFS HR 0.64; 95% CI 0.45–0.90; *p* = 0.011). (Fig. 5 and Supplementary Fig. 2).

#### Post-operative model

In the multivariable model incorporating post-operative factors (ypT, ypN, resection margin status, lymphatic vessel invasion (LVI), histological grade of differentiation, Mandard TRG and MMR status), MMR deficiency remained independently associated with improved outcome, conferring a 48% lower risk of death (OS HR 0.52; 95% CI 0.33–0.82; *p* = 0.005) and a 38% lower risk of progression (PFS HR 0.62; 95% CI 0.41–0.93, *p* = 0.022). (Fig. 5 and Supplementary Fig. 2).

## Discussion

In this large analysis of biomaterial from 1424 patients with resectable oesophagogastric adenocarcinoma treated within the MRC ST03 and OE05 trials, mismatch repair deficiency (MMRd) was identified in 5.5% of tumours and emerged as a strong, independent prognostic biomarker, extending previous observations to patients treated with perioperative chemotherapy [13, 15]. The 5.5% prevalence of MMRd in the current cohort is slightly lower than that reported in some gastric cancer series. However, it is comparable to reports of mixed gastroesophageal adenocarcinoma cohorts and remains substantially higher than that observed in pure oesophageal adenocarcinoma [16]. As the prevalence of MMRd is known to vary according to tumour location, disease stage, and study population, we believe these differences are at least partly explained by the composition of our cohort (28.2% gastric adenocarcinoma, 26.1% junctional, 45.6% oesophageal) rather than assay performance alone. Nevertheless, we cannot entirely exclude that a small number of MMRd cases may have been missed. If present, such misclassification would be expected to bias our results towards the null, making the observed differences between MMRd and MMRp tumours more conservative rather than artificially inflating them. Consistent with prior reports, MMRd was more common in older and female patients reflecting the known epidemiology of MMR deficiency in upper gastrointestinal tract cancer [6, 14].

Our results are supported by the post hoc analysis of the perioperatively treated patients from the CRITICS trial [23], and the individual patient data meta-analysis by Pietrantonio et al. [13]. In the CRITICS cohort, a comparable survival benefit was shown for patients with MSI-H tumours (5-year cancer-related survival 51.7% for patients with MSI-high tumours versus 38.6% for patients with EBV-negative/MSS tumours). Similarly, the meta-analysis by Pietrantonio et al., which pooled data from 1,556 patients enroled in MAGIC, CLASSIC, ARTIST, and ITACA-S trials, identified MSI/MMRd status as a favourable prognostic factor for both OS and PFS. Although higher absolute 5-year survival rates were observed in patients with MMRp or MMRd tumours in the meta-analysis, this likely reflects differences in patient composition. Approximately 63% of patients were of Asian origin, and the cohort was enriched for gastric cancers – e.g. populations with generally better outcome. In contrast, the ST03 and OE05 trials included a substantial proportion of patients with oesophageal and gastroesophageal junction cancers, who typically have a poorer prognosis. Importantly, our current study extends the existing evidence by validating the favourable prognostic impact of MMRd in one of the largest neoadjuvant/perioperative cohorts reported to date.

A persistent area of debate is whether cytotoxic chemotherapy may be detrimental in patients with MMRd oesophagogastric cancer treated with cytotoxic chemotherapy in the perioperative setting. Much of this concern originates from a post hoc analysis of the MAGIC trial [27], in which Smyth et al. reported that among patients who received perioperative chemotherapy, those with MSI-H tumours had inferior OS compared with patients with non–MSI-H tumours (HR 2.22; *p* = 0.04). In contrast, among patients treated with surgery alone, MSI-H status was associated with a non-significant trend toward improved survival relative to non–MSI-H (HR 0.35; *p* = 0.08). These findings have often been interpreted as evidence that patients with MMRd tumours derive little or no benefit from or may even be harmed by perioperative cytotoxic chemotherapy. However, these observations were exploratory and should be interpreted with caution given their *post hoc* nature and the very small sample sizes involved. The MSI subgroup was extremely small, only nine patients with MSI-H tumours had surgery alone and only ten patients with MSI-H tumours were treated by perioperative cytotoxic chemotherapy and surgery, rendering the estimates highly susceptible to imbalance from confounders. Moreover, the detrimental effect was inferred indirectly as patients with MSI-H tumours treated with perioperative chemotherapy were never formally compared to patients with MSI-H tumours managed with surgery alone.

The chemotherapy backbone also merits consideration. Patients included in the current analysis received platinum-based regimens in combination with fluoropyrimidines. While historical data in colorectal cancer suggest a reduced benefit from fluoropyrimidine monotherapy in patients with MMRd tumours [29], accumulating evidence indicates that the addition of oxaliplatin may mitigate this effect or even enhance treatment benefit in stage III patients with MMRd tumours, supporting our conclusions [30, 31].

Furthermore, in our analysis, MMRd was not associated with baseline clinical T or N stage, but MMRd was related to more favourable post-operative pathology following cytotoxic chemotherapy (lower rates of lymph node metastasis and a higher likelihood of R0 resection). Although this may reflect a degree of pathological sensitivity to treatment in patients with MMRd tumours, primary tumour regression according to the Mandard classification did not differ by MMR status. This observation is consistent with a separate retrospective study in localized oesophagogastric adenocarcinoma that likewise found no statistically significant difference in histological response by MMR status [32]. Taken together, the consistent survival advantage observed in patients with MMRd tumours alongside favourable postoperative pathological outcomes argues against a uniform detrimental effect of perioperative cytotoxic chemotherapy in patients with MMRd tumours.

Of note, although our study demonstrated similar post-progression survival in patients with MMRd and MMRp tumours, outcomes may differ in the contemporary treatment landscape, where immune checkpoint inhibitors are increasingly available for patients with immunotherapy-naïve MMRd tumours. Furthermore, the results of the MATTERHORN trial [33], the first phase III study to demonstrate a survival benefit with the addition of durvalumab to perioperative FLOT chemotherapy in patients with resectable gastric or gastroesophageal junction adenocarcinoma, provide a strong rationale for re-evaluating the prognostic and predictive significance of MMR status in patients receiving perioperative chemo-immunotherapy. In addition, the favourable pathological response rates observed with neoadjuvant nivolumab plus ipilimumab in the NEONIPIGA phase II study [34], support the potential role of neoadjuvant or perioperative combination immunotherapy strategy may be adopted for patients with resectable MMRd tumours. Nevertheless, access to combination immunotherapy remains limited in many healthcare systems. In such contexts, our findings suggest that neoadjuvant cytotoxic chemotherapy may still be associated with favourable survival in patients with MMRd tumours and therefore represents a clinically meaningful treatment option. Future prospective trials should directly compare perioperative immunotherapy alone with chemoimmunotherapy in patients with resectable MMRd tumours to establish the optimal treatment strategy.

In this study, we further explored outcomes according to the specific pattern of MMR protein loss which revealed heterogeneity within the MMRd subgroup. Tumours characterised by MLH1 loss, either alone or in combination with PMS2, accounted for the majority of MMRd cases and were associated with particularly favourable outcomes. In contrast, survival curves of patients with tumours with MSH2 or MSH6 loss overlapped with those of patients with MMRp tumours. Although these subgroup analyses are limited by small numbers, they suggest that MMRd tumours may not represent a biologically uniform entity with respect to prognosis. Differences in underlying aetiology (e.g. sporadic MLH1 loss versus Lynch-associated MSH2/MSH6 loss) may influence tumour biology and clinical behaviour. A retrospective study of a Danish colorectal cancer cohort also evaluated outcomes according to the pattern of MMR protein loss but found that improved survival was associated with MSH2/MSH6 loss rather than MLH1/PMS2 loss. Although these findings are noteworthy, they should be interpreted cautiously when extrapolating to our results in oesophagogastric cancer. In addition to differences in tumour type, the Danish study used a retrospective cohort and relied on propensity score matching, which reduced the analysable population [35]. Our observations require validation in larger cohorts with integrated molecular profiling. To better define the prognostic implications of specific MMR alterations, pathological reporting of the MMR status should include the specific protein(s) which show loss of expression.

This study has several strengths, including its large sample size derived from prospective clinical trial populations, comprehensive clinical data collection, mature survival data, and robust multivariable modelling incorporating both pre-operative and post-operative factors. Nevertheless, several limitations should be acknowledged. First, the absence of a surgery-alone treated comparative group precludes the assessment of the predictive value of MMR status for the benefit of perioperative chemotherapy. Second, the small number of patients in certain subgroups, such as those with isolated PMS2 loss limited statistical power for subgroup analyses. Third, because the cohort was derived from clinical trial populations, participants were younger and fitter than those encountered in routine clinical practice, which may limit the generalisability of the findings. Finally, the use of different chemotherapy regimens across the two trials may have introduced residual confounding despite adjustment for treatment regimen in multivariable models and trial specific subgroup analyses. Accordingly, these findings should be interpreted with appropriate caution and validated in larger, independent cohorts including real-world populations.

Based on our findings from the two largest UK neoadjuvant and perioperative chemotherapy trials in patients with resectable oesophagogastric cancer, there is no evidence that cytotoxic chemotherapy abrogates the favourable biology of MMRd tumours. Patients with MMRd tumours experienced improved OS and PFS compared to patients with MMRp tumours. This effect was consistent across multiple subgroup analyses and remained robust after adjustment for established pre-operative or post-operative prognostic factors. Importantly, the survival benefit associated with MMRd tumours was maintained among patients with high-risk prognostic factors at the time of resection and those who subsequently developed distant metastases. The prognostic impact of MMR deficiency was most pronounced among patients who underwent surgical resection, whereas there was no relationship between OS and MMR status in patients who did not proceed to surgery. This pattern suggests that the favourable biology of MMRd tumours is most relevant in the context of potentially curative treatment and may be attenuated in patients with rapidly progressive or unresectable disease. Overall, our findings do not support the hypothesis that peri‑operative chemotherapy is detrimental in patients with MMRd oesophagogastric cancer. Although high response rates have been reported with doublet immunotherapy regimens in phase 2 trials [36, 37], the addition of cytotoxic chemotherapy may still confer incremental benefit by increasing complete remission rates and enhancing the likelihood of organ preservation in selected patients.

## Supplementary information

Supplemental Material

## Data Availability

Data supporting the results reported in this manuscript can be obtained upon reasonable request from the corresponding authors.
