# Supplementary material for: The role of DNA mismatch repair status in oesophagogastric cancer treated with perioperative chemotherapy: evidence from the MRC ST03 and OE05 trials
Source: Br J Cancer. 2026 Aug 25;135(10):1731–42. doi: 10.1038/s41416-026-03594-9 (PMC13739907; doi:10.1038/s41416-026-03594-9)
Supplement: Supplementary file 1 [file 41416_2026_3594_MOESM1_ESM.docx]

The role of DNA mismatch repair status in oesophagogastric cancer treated with perioperative chemotherapy: evidence from the MRC ST03 and OE05 trials

Panagiotis Ntellas^1^, Tom Lund^2^, Georgina A Keogh^1^, Manuel Salto-Tellez^2,^ ^3^, Katharina von Loga^4^, Tatiany Silveira^2^, Susanna Riisnaes^2^, Jon Laye^5^, Ruth E Langley^6^, Matthew G Nankivell^6^, William H Allum^7^, Avani Athauda^1^, David Cunningham*^1^, Heike I Grabsch*^5, 8^

# **Supplementary Materials**

Table 1: Antibodies and staining conditions used for multiplex immunofluorescence

Table 2: REMARK checklist

Table 3: REMARK profile

Figure 1: Kaplan-Meier curves for overall survival and progression free survival by specific MMR protein loss pattern.

Figure 2. Multivariable analysis of pre- and post-operative predictors of progression-free survival.

## **Supplementary Table 1**. Antibodies and staining conditions used for multiplex immunofluorescence (mIF)

|  | Antibody 1 | Antibody 2 | Antibody 3 | Antibody 4 | Antibody 5 |
| --- | --- | --- | --- | --- | --- |
| Antigen | MLH1 | MSH2 | MSH6 | PMS2 | panCK |
| Manufacturer | DAKO | DAKO | DAKO | DAKO | DAKO |
| Clone | ES05 | FE11 | EP49 | EP51 | AE1/AE3 |
| Dilution | 1:100 | 1:200 | 1:50 | 1:100 | 1:200 |
| ER | ER2 | ER2 | ER1 | ER1 | ER1 |
| Opal | 520 | 620 | 570 | 690 | 780/TSA |
| Dilution | 1:150 | 1:150 | 1:150 | 1:150 | 1:50/1:75 |

## **Supplementary Table 2**. REMARK checklist

| **Item to be reported** | | **Page no.** |
| --- | --- | --- |
| **INTRODUCTION** | |  |
| 1 | State the marker examined, the study objectives, and any pre-specified hypotheses. | 7 |
| **MATERIALS AND METHODS** | |  |
| *Patients* | |  |
| 2 | Describe the characteristics (e.g., disease stage or co-morbidities) of the study patients, including their source and inclusion and exclusion criteria. | 8 |
| 3 | Describe treatments received and how chosen (e.g., randomised or rule-based). |  |
| *Specimen characteristics* | |  |
| 4 | Describe type of biological material used (including control samples) and methods of preservation and storage. | 9 |
| *Assay methods* | |  |
| 5 | Specify the assay method used and provide (or reference) a detailed protocol, including specific reagents or kits used, quality control procedures, reproducibility assessments, quantitation methods, and scoring and reporting protocols. Specify whether and how assays were performed blinded to the study endpoint. | 9-10 |
| *Study design* | |  |
| 6 | State the method of case selection, including whether prospective or retrospective and whether stratification or matching (e.g., by stage of disease or age) was used. Specify the time period from which cases were taken, the end of the follow-up period, and the median follow-up time. | 8-12 |
| 7 | Precisely define all clinical endpoints examined. |  |
| 8 | List all candidate variables initially examined or considered for inclusion in models. |  |
| 9 | Give rationale for sample size; if the study was designed to detect a specified effect size, give the target power and effect size. *(all available samples were analyzed)* |  |
| *Statistical analysis methods* | |  |
| 10 | Specify all statistical methods, including details of any variable selection procedures and other model-building issues, how model assumptions were verified, and how missing data were handled. | 10-12 |
| 11 | Clarify how marker values were handled in the analyses; if relevant, describe methods used for cutpoint determination. |  |
| **RESULTS** | |  |
| *Data* | |  |
| 12 | Describe the flow of patients through the study, including the number of patients included in each stage of the analysis (a diagram may be helpful) and reasons for dropout. Specifically, both overall and for each subgroup extensively examined report the numbers of patients and the number of events. | 12 |
| 13 | Report distributions of basic demographic characteristics (at least age and sex), standard (disease-specific) prognostic variables, and tumour marker, including numbers of missing values. | 12-13 |
| *Analysis and presentation* | |  |
| 14 | Show the relation of the marker to standard prognostic variables. | 13-14 |
| 15 | Present univariable analyses showing the relation between the marker and outcome, with the estimated effect (e.g., hazard ratio and survival probability). Preferably provide similar analyses for all other variables being analyzed. For the effect of a tumour marker on a time-to-event outcome, a Kaplan-Meier plot is recommended. | 14 |
| 16 | For key multivariable analyses, report estimated effects (e.g., hazard ratio) with confidence intervals for the marker and, at least for the final model, all other variables in the model. | 14-15 |
| 17 | Among reported results, provide estimated effects with confidence intervals from an analysis in which the marker and standard prognostic variables are included, regardless of their statistical significance. | 14-15 |
| 18 | If done, report results of further investigations, such as checking assumptions, sensitivity analyses, and internal validation. | 14-15 |
| **DISCUSSION** | |  |
| 19 | Interpret the results in the context of the pre-specified hypotheses and other relevant studies; include a discussion of limitations of the study. | 16-19 |
| 20 | Discuss implications for future research and clinical value. | 19-20 |

## **Supplementary Table 3**. REMARK profile

| Study marker | Remarks | | | |
| --- | --- | --- | --- | --- |
| Marker | Expression of mismatch repair proteins MLH1, MSH2, MSH6, PMS2 to determine DNA mismatch repair status | | | |
| Further variables | Treatment, performance status, BMI, sex, T status, N status, location of primary tumour, resection margin status, Mandard tumour regression grade, surgery status, grade of differentiation, type of progression, lymphovascular invasion status | | | |
| Patients | N | Remarks | | |
| Assessed for eligibility | 1960 | All patients randomised to the ST03 or OE05 trials | | |
| Excluded | 536 | No FFPE material received | | |
| Included | 1424 | (Oe05 trial: 736; ST03 trial: 688) | | |
| With outcome events | 907  955  907 | Overall survival: death from any cause  Progression-free survival  Post progression survival | | |
| Statistical analyses of survival outcomes | | | | |
| Analysis | Patients | Events | Variables considered | Results |
| **Overall survival** | | | | |
| A1: Univariable | 1424 | 907 | MMR-status | Figure 3A |
| A2: Multivariable pre-operative | 1346 | 853 | pre-operative | Figure 5A |
| A3: Multivariable post-operative | 1129 | 678 | post-operative | Figure 5B |
| **Progression-free survival** | | | | |
| B1: Univariable | 1424 | 955 | MMR-status | Figure 3B |
| B2: Multivariable pre-operative | 1346 | 897 | pre-operative | supplementary Figure 2A |
| B3: Multivariable post-operative | 1129 | 723 | post-operative | supplementary Figure 2B |
| **Post progression survival** | | | | |
| C1: Univariable | 955 | 907 | MMR-status | Figure 3C |
| C2: Multivariable | Not performed |  |  |  |

**Supplementary Figure 1. Kaplan-Meier curves for overall survival and progression free survival by specific MMR protein loss pattern.**

Top panel: Overall survival. Bottom panel : Progression free survival.

Patients with MLH1 loss in their tumour had the most favourable outcomes, whereas patients with isolated MSH2/MSH6 loss or PMS2 loss in their tumour had poorer outcomes which largely overlapped with the outcome of patients with MMR-proficient tumours. Cross groups log-rank comparisons were statistically significant for both OS (p=0.0041) and PFS (p=0.018).

**Supplementary Figure 2. Multivariable analysis of pre- and post-operative predictors of progression-free survival.**

**Top panel:** Pre-operative predictors of progression-free survival

**Bottom panel:** Post-operative predictors of progression-free survival.

Hazard ratio (HR) < 1 indicates lower risk of death or progression, HR > 1 indicates higher risk. Red bars represent statistically significant associations (p<0.05); grey bars indicate non-significant results (p≥0.05). *Reference category. ECX: epirubicin, cisplatin and capecitabine; Bev: bevacizumab; CF: cisplatin and 5-fluorouracil; T: depth of invasion; N: lymph node status; GOJ: gastroesophageal junction; BMI: body mass index; R1: resection margin positive; R0: resection margin negative; LVI: lymphovascular invasion; TRG: tumour regression grade of the primary tumour according to Mandard.
